# Dissemination of *Orientia tsutsugamushi* in a Case of Scrub Typhus

**DOI:** 10.4269/ajtmh.18-0689

**Published:** 2019-02

**Authors:** Yeon-Hee Han, Joo-Hee Hwang, Chang-Seop Lee

**Affiliations:** 1 Department of Nuclear Medicine, Chonbuk National University Medical School, Jeonju, Republic of Korea;; 2 Cyclotron Research Center, Molecular Imaging and Therapeutic Medicine Research Center, Chonbuk National University Medical School and Hospital, Jeonju, Republic of Korea;; 3 Department of Internal Medicine, Chonbuk National University Medical School, Jeonju, Republic of Korea;; 4 Research Institute of Clinical Medicine of Chonbuk National University-Biomedical Research Institute of Chonbuk National University Hospital, Jeonju, Republic of Korea

**Figure 1.**
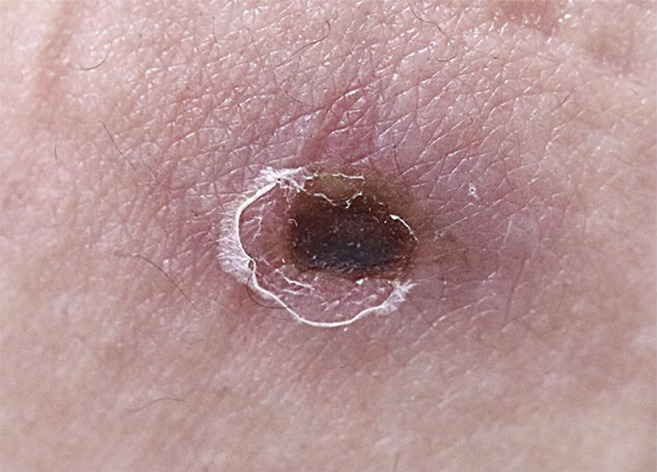
An eschar in a 22-year-old man. This figure appears in color at www.ajtmh.org.

**Figure 2.**
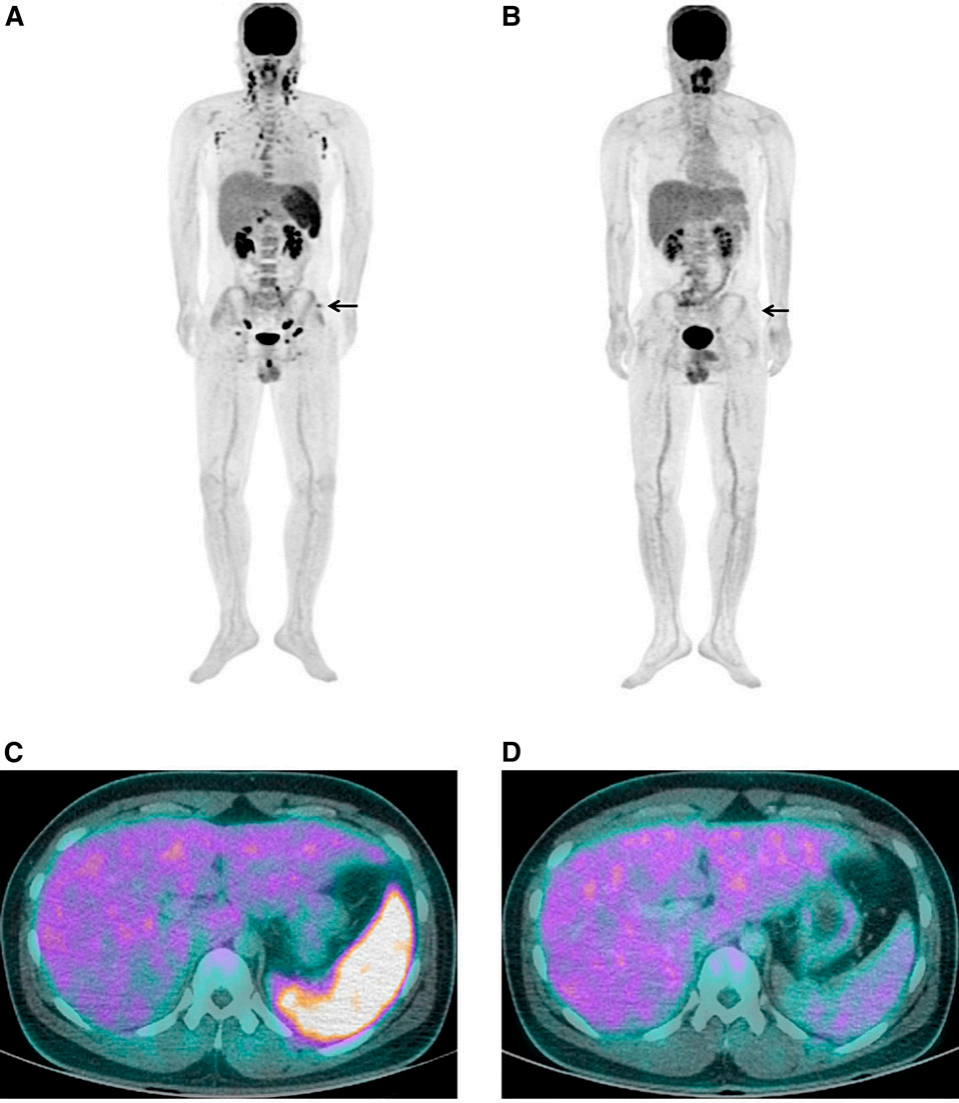
Generalized lymphadenopathy and hypermetabolism in the spleen of a 22-year-old man with scrub typhus. Fluorodeoxyglucose (FDG) positron emission tomography/computed tomography (PET/CT) image before (**A** and **C**) and after (**B** and **D**) treatment. Fluorodeoxyglucose PET/CT image shows a FDG uptake on the eschar lesion (arrow). This figure appears in color at www.ajtmh.org.

A 22-year-old man presented to the emergency room with fever, chills, headache, and skin rash that developed 7 days before. On physical examination, an eschar was observed on the left upper buttock (Figure 1). On F-18 fluorodeoxyglucose (FDG) positron emission tomography/computed tomography (PET/CT), FDG uptake was increased in the left upper buttock at the eschar site. Lymphadenopathy was observed in cervical, supraclavicular, axillary, mediastinum, intraperitoneal, retroperitoneal, iliac chain, and inguinal areas. Hepatosplenomegaly and hypermetabolism in the spleen were also observed (Figure 2A and C). Follow-up F-18 FDG PET/CT was taken around 3 weeks after antibiotic treatment. Fluorodeoxyglucose uptake was dramatically decreased in the eschar, lymph nodes, and spleen after treatment (Figure 2B and D).

A diagnosis of scrub typhus was confirmed by an increase in the indirect immunofluorescent antibody titer of 1:640. Nucleotide sequencing of a 56-kDa protein-encoding gene obtained from peripheral blood mononuclear cells was performed, and revealed the *Orientia tsutsugamushi* Boryong strain. Oral doxycycline 100 mg twice daily was administered for 7 days. The clinical course improved and the patient was stable 2 months after discharge.

Scrub typhus is the most common rickettsial disease in Korea and it occurs mainly in October and November.^1^ *Orientia tsutsugamushi* is the causative agent of scrub typhus and has tropism for dendritic cells and monocytes rather than endothelial cells.^2^ The infected dendritic cells and macrophages migrate from peripheral tissues via afferent lymphatic vessels into draining lymph nodes where they prime antigen-specific naive T cells.^3,4^ F-18 FDG PET/CT imaging showed the specific findings of scrub typhus as hypermetabolic eschar, lymphadenopathy, and splenomegaly. These findings could provide useful information for early diagnosis, and clinical response after treatments.
